# Burden of schizophrenia among Japanese patients: a cross-sectional National Health and Wellness Survey

**DOI:** 10.1186/s12888-022-04044-5

**Published:** 2022-06-18

**Authors:** Kenji Baba, Wenjia Guo, Yirong Chen, Tadashi Nosaka, Tadafumi Kato

**Affiliations:** 1 Medical Affairs, Sumitomo Pharma Co., Ltd., 13-1, Kyobashi 1-Chome, Chuo-ku, Tokyo, 104-8356 Japan; 2 Cerner Enviza, 83 Clemenceau Ave, Singapore, 239920 Singapore; 3 Department of Psychiatry and Behavioural Science, Juntendo University Graduate School of Medicine, 2 Chome-1-1 Hongo, Bunkyo City, Tokyo, 113-8421 Japan

**Keywords:** Schizophrenia, Prevalence, Health-related quality of life, Work productivity and activity impairment, Indirect cost

## Abstract

**Background:**

Schizophrenia places a great humanistic and financial burden to patients, families, and societies, and the burden is substantially impacted by comorbid conditions. This study aimed to estimate the lifetime prevalence of schizophrenia and to assess the health-related quality of life (HRQoL), work productivity, and indirect cost among schizophrenia patients with and without comorbidities (depressive symptoms, sleep disturbances, and anxiety problems).

**Methods:**

This is a secondary analysis of existing data collected in 2019 from the Japan National Health and Wellness Survey. The schizophrenia patients were categorized based on their Patient Health Questionnaire-9 score, self-reported experience of sleep disturbances, and anxiety problems. The lifetime prevalence was estimated using the total number of diagnosed schizophrenia patients as the numerator and the total number of respondents as the denominator. The HRQoL was evaluated through the Short Form 12-Item (version 2) Health Survey and EuroQoL 5-dimensions scale. Work productivity and annual indirect costs were evaluated through the Work Productivity and Activity Impairment instrument and monthly wage rates. Multivariate analyses included the comparison of outcomes using generalized linear models.

**Results:**

The study was conducted with 178 schizophrenia patients with an average age of 42.7 years old and an estimated lifetime prevalence of 0.59% (95% CI: 0.51%, 0.68%). Patients who experienced sleep disturbances, more severe depressive symptoms, and anxiety problems had lower HRQoL, higher levels of absenteeism, presenteeism, total work productivity and activity impairment, and almost twice more indirect costs, compared to those without these conditions.

**Conclusion:**

Comorbid conditions among patients with schizophrenia impact significantly on their quality of life, work productivity as well as indirect costs.

**Supplementary Information:**

The online version contains supplementary material available at 10.1186/s12888-022-04044-5.

## Background

The schizophrenia global data evidenced an estimated median lifetime prevalence of 0.48% [1], with an onset in adolescence and young adulthood [2]. It is estimated that 70.8% (or 14.8 million) of the schizophrenia cases occur in the 25–54 years age group with a prevalence peak at around 40 years old [2]. In Japan, lifetime prevalence of schizophrenia was estimated to be between 0.19 to 1.79%, based on epidemiological surveys between 1940s to 1970s [3]. It is worth noting that those epidemiological surveys summarized by *Nakane* et al. was focused on locations with unique social environments, particularly isolated and remote communities, which could impose limitations on the interpretation and generalizability of the results [3]. It has also been recently reported in Japan that nearly one-third of all long-term hospitalized psychiatric patients were aged ≥75 years old and two-third of all long-term hospitalized psychiatric patients were schizophrenia patients [4]. Therefore, an assessment of schizophrenia prevalence based on a broader patient population is needed to provide a current estimation of the prevalence and to understand the burden of disease in Japan.

Compared with the general population, life expectancy for patients with schizophrenia is reduced with a weighted average of 14.5 years of potential life lost [5]. This reduction in life expectancy is likely owing to depressive symptoms and physical morbidities (i.e., cardiovascular disease and type II diabetes mellitus). Specifically, a cohort study has shown a relationship between depressive symptoms and suicide risk [6], and a systematic review has shown that high suicide risk is one of the major causes of shortened life expectancy in schizophrenia patients [7]. In addition to a shortened life expectancy, schizophrenia contributes 13.4 (95% uncertainty interval: 9.9–16.7) million years of life lived with disability (YLDs) to the burden of disease, equivalent to 1.7% of total YLDs globally in 2016 which is considered substantial [2].

Schizophrenia also places significant financial burden on families, health systems, and society [8, 9]. In Japan, it was estimated that the total cost of schizophrenia was approximately JPY (¥)2.77 trillion (US$23.7 billion). The greatest part of that cost (72% of the total cost) was contributed by the indirect cost (including morbidity and mortality costs). The direct costs, morbidity, and mortality costs were estimated to be ¥0.77 trillion ($6.59 billion), ¥1.85 trillion ($15.8 billion) and ¥0.16 trillion ($1.33 billion), respectively [10].

Regarding the costs of informal care (care provided by non-professional caregivers, such as family, friends, acquaintances, or neighbors), caregivers of patients with schizophrenia are largely affected by the burden of the disease. They had significantly higher work impairment than those caregivers for other conditions [11]. In Japan, 19% of caregivers reported that they used to work but had to quit their job and gave up an income of approximately JPY 1.3 million because of caregiving [12]. For those who are still in the workforce, their productivity was also impacted. About 5% of them reported missing work due to taking care of schizophrenia patients in the last 7 days while their productivity was reduced by approximately 25%. Among total annual productivity loss, JPY 2.36 million was due to reduced work performance (presenteeism) while at work and JPY 197,355 is due to sick leave (absenteeism) [12].

The humanistic burden among patients with schizophrenia is substantially impacted by comorbid depressive and anxiety symptoms, cognitive impairment, and caregiver burden [9, 13, 14]. Comorbid depression and anxiety were found to negatively impact the progression, morbidity, and mortality of schizophrenia. In addition, these factors could be an obstacle to recovery or remission [9, 13, 14]. The reported prevalence of comorbid depression and anxiety among patients with schizophrenia is 32.6% (depression) [15] and 38.3% (anxiety) [16]. A higher prevalence of anxiety and depression is correlated with lower quality of life (QoL) [13, 17].

The QoL of schizophrenia patients is worse than the general population and worse than many patients with physical and other mental disorders [18]. Besides, the quality of life and ability to engage in everyday activities are reduced in schizophrenia patients due to significant social and/or occupational dysfunction, which impose a significant caregiver burden [12]. In Japan, however, epidemiological surveys of schizophrenia patients have not been conducted in recent years. Hence, the prevalence of schizophrenia, its impact, and indirect costs among those patients have not been well studied. Through utilizing a large-scale self-reported online survey, the objective of our study was to estimate the prevalence of schizophrenia and to assess the health-related quality of life (HRQoL), work productivity and activity impairment (WPAI), and indirect cost among adult patients with a self-reported physician diagnosis of schizophrenia with and without comorbidities. The comorbidities of interest in this study include depressive symptoms, sleep disturbances, and anxiety problems.

## Methods

### Study design and participants

The study is a secondary analysis of existing data collected in 2019 from the cross-sectional Japan National Health and Wellness Survey (NHWS). The NHWS is a self-administered online survey covering self-reported information on respondent characteristics, disease status, and health outcomes, administered to adult respondents (aged 18 and above) in Japan and 11 other countries.

In Japan, a stratified random sampling framework with quotas approximating the age and gender distribution according to the Japanese census data was implemented to ensure that the NHWS respondents were representative of the general adult population in Japan. The NHWS was granted exemption status upon review by Pearl International Review Board (Indianapolis, IN). All respondents provided informed consent prior to participation. Anonymized data were utilized in this study, and the Juntendo University Ethics Committee assessed and determined that institutional ethics approval could be waived, according to the Ministry of Education, Culture, Sports, Science, and Technology and the Ministry of Health, Labour, and Welfare’s ethical guidelines for medical and health research involving human subjects [19].

### Study population

Respondents who self-reported a physician diagnosis of schizophrenia were included in this study. Among diagnosed schizophrenia patients, they were divided into subgroups based on their Patient Health Questionnaire (PHQ)-9 score [20], the self-reported experience of sleep disturbances, as well as self-reported experience of anxiety problems.

### Measures

Demographic characteristics analyzed in this study included age, gender, marital status, level of education, employment status, household income, geographic location, and health insurance status. The general health characteristics included body mass index (BMI), Charlson Comorbidity Index (CCI) [21, 22], smoking status, alcohol use, and exercise behavior. The healthcare resources utilization (HCRU) was assessed as the number of healthcare provider (HCP) visits, the number of emergency room (ER) visits, and the number of hospitalizations in the past 6 months for own medical conditions, as well as the out-of-pocket payment for own prescription medications in an average month.

#### Health-related quality of life (HRQoL)

The HRQoL was measured using the Short Form 12-Item (version 2) Health Survey (SF-12v2) instrument [23]. This is a multipurpose, generic instrument comprising of 12 questions. The SF-12v2 instrument has been translated and validated for use in the Japanese population [24, 25]. A Japan-specific norm-based scoring algorithm was applied to derive three summary scores: mental component summary (MCS), physical component summary (PCS), and role component summary (RCS) scores [24]. Summary scores range from 0 to 100, with higher scores indicating better quality of life (QoL). The EuroQoL 5-dimensions scale (EQ-5D-5L) summary index was also used to quantify health state utilities with higher scores indicating better QoL [26]. The EQ-5D also has a visual analog scale (VAS) where respondents indicated their self-rated health on a line from 0 (“best imaginable health”) to 100 (“worst imaginable health state”).

#### Impact on work-related activities

The Work Productivity and Activity Impairment (WPAI) is a six-item validated instrument consisting of four metrics in the form of percentages: absenteeism (the percentage of work time missed because of one’s health in the past 7 days), presenteeism (the percentage of impairment experienced because of one’s health while at work in the past 7 days), total work productivity impairment (an overall impairment estimate that is a combination of absenteeism and presenteeism), and activity impairment (the percentage of impairment in daily activities because of one’s health in the past 7 days). Higher percentage values indicate greater impairment. Respondents who reported being employed full-time, part-time or self-employed were surveyed for absenteeism, presenteeism, and total work productivity impairment. All respondents provided data for activity impairment [27].

#### Indirect costs

Indirect costs in this study only included morbidity cost. The annual indirect costs were calculated by integrating information from the WPAI and monthly wage rates from the Japan Basic Survey on Wage Structure, 2019 [28] using the human-capital method [29]. Total indirect costs due to work productivity impairment were calculated.

#### The severity of depressive symptoms

Self-reported severity of depressive symptoms was assessed by the validated PHQ-9 questionnaire [20]. It is a 9-item questionnaire that measures the frequency of depression symptoms, with items scored on a 4-point scale (not at all = 0 to nearly every day = 3). Two cut-off points (10 and 14) were used for categorization based on depression severity [30, 31].

#### Sleep disturbances and anxiety problems

Experience of sleep disturbances (including insomnia, narcolepsy, sleep apnea, and other sleep difficulties) and anxiety problems (including anxiety symptoms, generalized anxiety disorder, obsessive-compulsive disorder, panic disorder, phobias, post-traumatic stress disorder, and social anxiety disorder) were self-reported by all respondents in NHWS.

### Statistical analysis

The lifetime prevalence of schizophrenia in Japan was estimated using the total number of diagnosed schizophrenia patients as the numerator and the total number of NHWS respondents in Japan as the denominator. Demographic and general health characteristics, as well as HCRU, were reported using counts and percentages for categorical variables and means and standard deviations (SDs) for continuous variables, among patients with schizophrenia. Bivariate comparisons between the subgroups concerning these characteristics and health outcomes were conducted using independent t-tests for continuous variables and chi-square tests for categorical variables.

Multivariate analyses included the comparison of outcomes (HRQoL, WPAI, indirect costs) using generalized linear models (GLMs) to control for covariates between schizophrenia patients with different severity of depressive symptoms. GLMs with identity link functions were used for approximately normally distributed outcomes (including HRQoL summary scores, EQ-5D index, and VAS); while GLMs with log link functions were used for outcome variables with skewed distribution (including WPAI measures and indirect costs). Age, gender, region, marital status, level of education, employment status, household income, insurance type, BMI, CCI, smoking status, alcohol use, exercise behavior, and out-of-pocket payment were included in the GLMs as covariates. Estimated adjusted means with 95% confidence intervals (CIs) and *p*-values for all outcomes were reported. Association between the PHQ-9 categories (< 5, 5–9, 10–14, 15–19, and ≥ 20) and patient health outcomes among schizophrenia patients were further investigated using Kruskal-Wallis H Test. This approach for analysing NHWS data has previously been described in other studies [32].

All statistical analyses were performed using IBM SPSS Statistics Version 25 [33]. *P*-values of less than 0.05 were considered statistically significant.

## Results

### Participants and lifetime prevalence

Among the 30,006 respondents to the 2019 Japan NHWS, 178 participants self-reported a diagnosis of schizophrenia. The lifetime prevalence was estimated to be 0.59% (95% CI: 0.51, 0.68%). Among diagnosed schizophrenia patients, 93 scored less than 10 using PHQ-9 (PHQ-9 < 10) while 85 scored at least 10 using PHQ-9 (PHQ-9 ≥ 10); 117 scored less than 14 (PHQ-9 < 14) while 61 scored at least 14 (PHQ-9 ≥ 14). About half (*N* = 88) of schizophrenia patients experienced sleep disturbances, and 90 had not experienced any sleep disturbances. Concerning the anxiety problems, 66 patients with schizophrenia experienced this problem while 112 did not.

### Demographic and health characteristics

The average age of schizophrenia patients was 42.70 and almost 90% of patients were below 65 years old. There were similar proportions of males and females among patients with schizophrenia. A small proportion of patients (29.2%) were married or living with a partner, and 32.6% of schizophrenia patients had a university degree. More than half of the patients were employed and about one-third of the patients were overweight (Table 1).

**Table 1:** Demographic and general health characteristics, healthcare resource utilization of schizophrenia patients in Japan

	Schizophrenia patients (***N*** = 178)	
**Continuous Variables**	**Mean**	**SD**
Age	42.70	14.38
Charlson Comorbidity Index	0.55	2.86
Healthcare resource utilisation		
*No. of physician visits in the past 6 months*	12.12	12.12
*No. of ER visits in the past 6 months*	0.12	0.75
*No. of hospitalizations in the past 6 months*	0.24	1.06
**Categorical Variables**	**n**	**%**
Age group		
*18–64*	159	89.3%
*≥ 65*	19	10.7%
Gender		
*Male*	89	50.0%
*Female*	89	50.0%
Marital Status		
*Married or living with partner*	52	29.2%
*Not married/Decline to answer*	126	70.8%
Level of Education		
*University degree*	58	32.6%
*Not/Decline to answer*	120	67.4%
Employment Status		
*Currently employed*	91	51.1%
*Not*	87	48.9%
Household Income		
*< ¥3,000,000*	70	39.3%
*¥3,000,000 to < ¥5,000,000*	44	24.7%
*¥5,000,000 to < ¥8,000,000*	24	13.5%
*¥8,000,000 or more*	18	10.1%
*Decline to answer*	22	12.4%
Body Mass Index		
*Underweight (BMI < 18.5)*	21	11.8%
*Normal (BMI ≥ 18.5 & < 25)*	93	52.2%
*Overweight (BMI ≥ 25)*	57	32.0%
*Decline to answer*	7	3.9%
Smoking Status		
*Never*	92	51.7%
*Former*	40	22.5%
*Current*	46	25.8%
Alcohol Use		
*≤ once per week*	143	80.3%
*≥ 2–3 times per week*	35	19.7%
Vigorous Exercise in Past 30 Days		
*0–11 times*	151	84.8%
*≥ 12 times*	27	15.2%
Insurance type		
*National health insurance*	89	50.0%
*Social insurance*	56	31.5%
*Late stage elderly insurance*	7	3.9%
*Other/No insurance*	26	14.6%
Out of pocket payment for own prescription medications in an average month		
*¥0*	35	19.7%
*¥1 to ¥999*	25	14.0%
*¥1000 to ¥1999*	21	11.8%
*¥2000 to ¥2999*	16	9.0%
*¥3000 to ¥4999*	24	13.5%
*¥5000 to ¥9999*	17	9.6%
*¥10,000 to ¥49,999*	11	6.2%
*¥50,000 to ¥99,999*	1	0.6%
*¥100,000 or more*	3	1.7%
*Don’t know*	25	14.0%
Region		
*Hokkaido*	8	4.5%
*Tohoku*	16	9.0%
*Kanto*	57	32.0%
*Chubu*	26	14.6%
*Kansai/Kinki*	34	19.1%
*Chugoku*	13	7.3%
*Shikoku*	7	3.9%
*Kyushu/Okinawa*	17	9.6%

### Diagnosed schizophrenia patients with milder versus more severe depressive symptoms

Demographic and general health characteristics were compared between Japanese schizophrenia patients with milder (PHQ-9 < 10) or more severe depressive symptoms (PHQ-9 ≥ 10). Among the patient characteristics examined, significant differences were only observed in age and marital status – patients with more severe depressive symptoms were younger (38.80 vs. 46.26) and fewer of them were married or living with a partner (20.0% vs. 37.6%) (Table 2).

**Table 2:** Demographic and general health characteristics of schizophrenia patients with PHQ-9 < 10 vs. PHQ-9 ≥ 10 in Japan

	Schizophrenia patients and PHQ-9 < 10 ***N*** = 93		Schizophrenia patients and PHQ-9 ≥ 10 ***N*** = 85		
**Continuous Variables**	**Mean**	**SD**	**Mean**	**SD**	***P*-value**
Age	46.26	14.38	38.80	13.40	< 0.001
Charlson Comorbidity Index	0.59	3.48	0.51	2.00	0.843
Healthcare resource utilisation					
*No. of physician visits in the past 6 months*	9.65	9.76	14.84	13.81	0.004
*No. of ER visits in the past 6 months*	0.14	0.95	0.11	0.44	0.764
*No. of hospitalizations in the past 6 months*	0.26	1.33	0.22	0.66	0.830
**Categorical Variables**	**n**	**%**	**n**	**%**	***P*-value**
Age group					
*18–64*	80	86.0%	79	92.9%	0.135
*≥65*	13	14.0%	6	7.1%	
Gender					
*Male*	46	49.5%	43	50.6%	0.881
*Female*	47	50.5%	42	49.4%	
Marital Status					
*Married or living with partner*	35	37.6%	17	20.0%	0.010
*Not married/Decline to answer*	58	62.4%	68	80.0%	
Level of Education					
*University degree*	31	33.3%	27	31.8%	0.824
*Not/Decline to answer*	62	66.7%	58	68.2%	
Employment Status					
*Currently employed*	49	52.7%	42	49.4%	0.662
*Not*	44	47.3%	43	50.6%	
Household Income					
*< ¥3,000,000*	38	40.9%	32	37.6%	0.665
*¥3,000,000 to < ¥5,000,000*	22	23.7%	22	25.9%	
*¥5,000,000 to < ¥8,000,000*	15	16.1%	9	10.6%	
*¥8,000,000 or more*	9	9.7%	9	10.6%	
*Decline to answer*	9	9.7%	13	15.3%	
Body Mass Index					
*Underweight (BMI < 18.5)*	13	14.0%	8	9.4%	0.679
*Normal (BMI ≥ 18.5 & < 25)*	45	48.4%	48	56.5%	
*Overweight (BMI ≥ 25)*	31	33.3%	26	30.6%	
*Decline to answer*	4	4.3%	3	3.5%	
Smoking Status					
*Never*	50	53.8%	42	49.4%	0.581
*Former*	22	23.7%	18	21.2%	
*Current*	21	22.6%	25	29.4%	
Alcohol Use					
*≤ once per week*	74	79.6%	69	81.2%	0.788
*≥ 2–3 times per week*	19	20.4%	16	18.8%	
Vigorous Exercise in Past 30 Days					
*0–11 times*	78	83.9%	73	85.9%	0.709
*≥ 12 times*	15	16.1%	12	14.1%	
Insurance type					
*National health insurance*	42	45.2%	47	55.3%	0.142
*Social insurance*	33	35.5%	23	27.1%	
*Late stage elderly insurance*	6	6.5%	1	1.2%	
*Other/No insurance*	12	12.9%	14	16.5%	
Out of pocket payment for own prescription medications in an average month					
*¥0*	16	17.2%	19	22.4%	0.093
*¥1 to ¥999*	18	19.4%	7	8.2%	
*¥1000 to ¥1999*	15	16.1%	6	7.1%	
*¥2000 to ¥2999*	6	6.5%	10	11.8%	
*¥3000 to ¥4999*	11	11.8%	13	15.3%	
*¥5000 to ¥9999*	11	11.8%	6	7.1%	
*¥10,000 to ¥49,999*	3	3.2%	8	9.4%	
*¥50,000 to ¥99,999*	0	0.0%	1	1.2%	
*¥100,000 or more*	1	1.1%	2	2.4%	
*Don’t know*	12	12.9%	13	15.3%	
Region					
*Hokkaido*	5	5.4%	3	3.5%	0.714
*Tohoku*	8	8.6%	8	9.4%	
*Kanto*	29	31.2%	28	32.9%	
*Chubu*	11	11.8%	15	17.6%	
*Kansai/Kinki*	21	22.6%	13	15.3%	
*Chugoku*	7	7.5%	6	7.1%	
*Shikoku*	5	5.4%	2	2.4%	
*Kyushu/Okinawa*	7	7.5%	10	11.8%	

The bivariate analysis demonstrated that Japanese norm-based MCS and RCS scores of the SF-12v2 were lower in patients with schizophrenia and with PHQ-9 ≥ 10, compared to schizophrenia patients with PHQ-9 < 10 (*p* <  0.001). No significant differences were observed in PCS score (Table 3). In addition, lower EQ-5D utility scores and VAS scores were reported in schizophrenia patients with PHQ-9 ≥ 10 compared to those with PHQ-9 < 10 (both *p* <  0.001). Comparing to patients with PHQ-9 < 10, schizophrenia patients with PHQ-9 ≥ 10 had higher levels of impairment in terms of absenteeism, presenteeism, total work productivity impairment, activity impairment and higher indirect costs (all *p* <  0.05).

**Table 3:** Unadjusted and adjusted means of health outcomes for schizophrenia patients with PHQ-9 < 10 vs. PHQ-9 ≥ 10 in Japan

	Schizophrenia patients and PHQ-9 < 10 ***N*** = 93			Schizophrenia patients and PHQ-9 ≥ 10 ***N*** = 85			
**Health outcomes (Unadjusted)**	**n**	**Mean**	**SD**	**n**	**Mean**	**SD**	***P*-value**
HRQoL							
*MCS (Japanese norm)*	93	49.97	11.22	85	40.60	9.80	< 0.001
*PCS (Japanese norm)*	93	47.41	12.20	85	44.90	15.76	0.235
*RCS (Japanese norm)*	93	41.94	15.55	85	25.10	15.25	< 0.001
*EQ-5D index*	93	0.80	0.16	85	0.60	0.18	< 0.001
*EQ-5D VAS*	93	68.18	21.71	85	43.34	24.57	< 0.001
WPAI							
*Absenteeism %*	47	11.17	19.17	40	24.28	33.01	0.024
*Presenteeism %*	49	26.73	29.11	39	60.51	26.15	< 0.001
*Total work impairment %*	47	33.79	30.57	38	65.87	27.32	< 0.001
*Total activity impairment %*	93	28.06	26.14	85	61.18	26.16	< 0.001
Indirect cost (in thousand yen)	47	1261.45	1173.17	38	2418.39	1203.98	< 0.001
**Health outcomes (Adjusted)**	**n**	**Mean**	**SE**	**n**	**Mean**	**SE**	***P*-value**
HRQoL							
*MCS (Japanese norm)*	93	50.45	2.07	85	41.75	2.10	< 0.001
*PCS (Japanese norm)*	93	49.50	2.51	85	44.15	2.55	0.006
*RCS (Japanese norm)*	93	40.09	2.89	85	26.28	2.94	< 0.001
*EQ-5D index*	93	0.78	0.03	85	0.56	0.03	< 0.001
*EQ-5D VAS*	93	67.77	4.55	85	44.29	4.63	< 0.001
WPAI							
*Absenteeism %*	47	1.08	0.86	40	14.22	11.85	0.001
*Presenteeism %*	49	14.53	4.39	39	38.26	13.04	< 0.001
*Total work impairment %*	47	25.75	7.33	38	58.38	19.83	0.004
*Total activity impairment %*	93	22.82	4.26	85	53.55	10.17	< 0.001
Indirect cost (in thousand yen)	47	892.76	270.62	38	1965.09	711.39	0.010

After adjusting for age, gender, region, marital status, level of education, employment status, household income, insurance type, BMI, CCI, smoking status, alcohol use, exercise behavior and out of pocket payment, schizophrenia patients with PHQ-9 ≥ 10 had significantly lower MCS (41.75 vs. 50.45, *p* <  0.001), PCS (44.15 vs. 49.50, *p* = 0.006) and RCS (26.28 vs. 40.09, *p* <  0.001) scores, lower EQ-5D utility score (0.56 vs. 0.78, *p* <  0.001) and VAS score (44.29 vs. 67.77, *p* <  0.001), compared to schizophrenia patients with PHQ-9 < 10 (Table 3). In terms of work productivity, after adjustment, schizophrenia patients with PHQ-9 ≥ 10 had significantly higher absenteeism (14.22% vs. 1.08%, *p* = 0.001), presenteeism (38.26% vs. 14.53%, *p* <  0.001), total work productivity impairment (58.38% vs. 25.75%, *p* = 0.004) and activity impairment (53.55% vs. 22.82%, *p* <  0.001), compared to schizophrenia patients with PHQ-9 < 10. Indirect costs due to work productivity impairment were significantly higher for patients with PHQ-9 ≥ 10 than those with PHQ-9 < 10 (1965.1 vs. 892.8 thousand-yen, *p* = 0.010).

Comparing Japanese schizophrenia patients with PHQ-9 < 14 and PHQ-9 ≥ 14, similar findings were observed (Supplementary Tables 1 and 2).

Further investigation on the association between PHQ-9 and the health outcomes also confirmed that the PHQ-9 score was significantly associated with health outcomes among patients with schizophrenia (Table 4). Specifically, MCS and EQ-5D index scores decreased while presenteeism and total work productivity impairment increased across the PHQ-9 score categories (Supplementary Fig. 1).

**Table 4:** Association between PHQ-9 and health outcomes among schizophrenia patients in Japan

	PHQ-9 groups										
	< 5		5–9		10–14		15–19		≥20		
**Health outcomes**	**n**	**Mean**	**n**	**Mean**	**n**	**Mean**	**n**	**Mean**	**n**	**Mean**	***P*-value**
HRQoL											
*MCS (Japanese norm)*	54	52.61	39	46.32	30	42.97	23	41.23	32	37.93	< 0.001
*PCS (Japanese norm)*	54	48.98	39	45.23	30	45.44	23	42.58	32	46.07	0.365
*RCS (Japanese norm)*	54	42.93	39	40.58	30	29.60	23	30.15	32	17.25	< 0.001
*EQ-5D index*	54	0.84	39	0.74	30	0.64	23	0.63	32	0.53	< 0.001
*EQ-5D VAS index*	54	72.15	39	62.69	30	49.23	23	49.65	32	33.28	< 0.001
WPAI											
*Absenteeism %*	31	12.71	16	8.19	16	17.13	10	31.10	14	27.57	0.112
*Presenteeism %*	32	21.56	17	36.47	16	50.00	9	57.78	14	74.29	< 0.001
*Overall work impairment %*	31	30.74	16	39.69	15	55.53	9	64.67	14	77.71	< 0.001
*Activity impairment %*	54	20.37	39	38.72	30	54.33	23	55.22	32	71.88	< 0.001
Indirect cost (in thousand yen)	31	1201.64	16	1377.33	15	2152.31	9	2424.51	14	2699.53	0.002

### Diagnosed schizophrenia patients with experience of sleep disturbances versus without

No significant differences in terms of baseline patient characteristics were observed between schizophrenia patients with and without experience of sleep disturbances (Supplementary Table 3). Patients with experience of sleep disturbances had significantly more physician visits and hospitalizations in the past 6 months than patients without experience of sleep disturbances.

Bivariate comparisons between schizophrenia patients with and without experience of sleep disturbances demonstrated that those who experienced sleep conditions had lower MCS (43.02 vs. 47.91, *p* = 0.004) and RCS (29.14 vs. 38.56, *p* <  0.001) scores, EQ-5D index (0.65 vs. 0.75, *p* <  0.001) and VAS (46.64 vs. 65.79, *p* <  0.001) scores compared to patients without experience of sleep disturbances (Table 5). Schizophrenia patients with experience of sleep disturbances also had significantly higher absenteeism (26.11% vs. 7.20%, *p* = 0.001), presenteeism (53.11% vs. 29.77%, *p* = 0.001), total work productivity impairment (63.16% vs. 32.00%, *p* <  0.001) and total activity impairment (53.30% vs. 34.67%, *p* <  0.001), as well as significantly higher indirect cost (2370.0 vs. 1144.1 thousand yen, *p* <  0.001).

**Table 5:** Unadjusted means of health outcomes of schizophrenia patients with and without experience of sleep disturbances and anxiety problems in Japan

	Schizophrenia patients with experience of sleep disturbances ***N*** = 88 (1)			Schizophrenia patients without experience of sleep disturbances ***N*** = 90 (2)			Schizophrenia patients with experience of anxiety problems ***N*** = 66 (3)			Schizophrenia patients without experience of anxiety problems ***N*** = 112 (4)			***P***-value	
**Health outcomes (Unadjusted)**	**n**	**Mean**	**SD**	**n**	**Mean**	**SD**	**n**	**Mean**	**SD**	**n**	**Mean**	**SD**	**(1) vs. (2)**	**(3) vs. (4)**
HRQoL														
*MCS (Japanese norm)*	88	43.02	11.49	90	47.91	11.11	66	43.71	11.57	112	46.55	11.43	0.004	0.113
*PCS (Japanese norm)*	88	44.43	15.27	90	47.95	12.55	66	44.25	16.24	112	47.37	12.48	0.094	0.152
*RCS (Japanese norm)*	88	29.14	16.85	90	38.56	16.99	66	26.90	16.77	112	38.03	16.69	< 0.001	< 0.001
*EQ-5D index*	88	0.65	0.21	90	0.75	0.17	66	0.62	0.22	112	0.75	0.16	< 0.001	< 0.001
*EQ-5D VAS*	88	46.64	27.36	90	65.79	21.21	66	44.12	28.44	112	63.51	21.92	< 0.001	< 0.001
WPAI														
*Absenteeism %*	46	26.11	31.10	41	7.20	17.21	35	21.94	23.64	52	14.00	28.97	0.001	0.181
*Presenteeism %*	45	53.11	33.36	43	29.77	26.95	36	53.61	33.05	52	33.46	29.56	0.001	0.004
*Total work impairment %*	44	63.16	29.75	41	32.00	28.97	35	63.66	27.98	50	37.26	32.34	< 0.001	< 0.001
*Total activity impairment %*	88	53.30	31.32	90	34.67	27.69	66	55.91	31.57	112	36.79	28.32	< 0.001	< 0.001
PHQ-9 score	88	13.44	8.23	90	7.73	7.54	66	14.73	9.02	112	8.10	6.90	< 0.001	< 0.001
Indirect cost (in thousand yen)	44	2369.98	1281.20	41	1144.09	1031.88	35	2321.00	1194.38	50	1399.03	1270.28	< 0.001	0.001

GLMs were not used for adjustment as the baseline characteristics were similar between the two groups.

### Diagnosed schizophrenia patients with experience of anxiety problems versus without

No significant differences in terms of baseline patient characteristics were observed between schizophrenia patients with and without experience of anxiety problems, except for CCI (Supplementary Table 3). Patients with experience of anxiety problems had significantly more physician visits and hospitalizations in the past 6 months compared to patients without experience of anxiety problems.

In the bivariate comparisons, schizophrenia patients with experience of anxiety problems experienced significantly lower RCS score (26.90 vs. 38.03, *p* <  0.001), as well as EQ-5D index (0.62 vs. 0.75, *p* <  0.001) and VAS (44.12 vs. 63.51, *p* <  0.001) scores. In addition, presenteeism (53.61% vs. 33.46%, *p* = 0.004), total work productivity impairment (63.66% vs. 37.26%, *p* <  0.001), and total activity impairment (55.91% vs. 36.79%, *p* <  0.001), as well as indirect cost (2321.0 vs. 1399.0 thousand yen, *p* = 0.001), were significantly higher among schizophrenia patients with experience of anxiety problems compared to those without experience of anxiety problems.

GLMs were not used for adjustment as the baseline characteristics were similar between the two groups.

### National morbidity cost of schizophrenia disorder in Japan

It is also worth mentioning that despite the average age (43 years old) of the patients with schizophrenia in Japan, about half were not currently employed. This contributes to the total morbidity cost of schizophrenia in Japan, in addition to the indirect cost incurred by impairment of work productivity for those who are employed. As post-hoc exploratory analysis, the national morbidity cost of schizophrenia was estimated to be 1074 billion Japanese yen, using available data from this study and government-published employment and income data (details of the methodology are described in Supplementary material 2). Cost due to unemployment contributed to more than half of total morbidity cost (552 billion yen; 51.4%).

## Discussion

### Lifetime prevalence and indirect cost

Our findings demonstrate an estimated schizophrenia lifetime prevalence of 0.59% (95% CI: 0.51%, 0.68%). Local data are aligned to ours as they detected a lifetime prevalence in Japan ranging from 0.19 to 1.79% [3]. Also, *Goldner* et al., through a global systematic literature review, found a similar lifetime prevalence of 0.55% [34]. It is important to notice that the data have a 6- to 14-fold variation across geographical regions that may be attributed to the different populations’ characteristics (i.e., age, urbanicity, different risks factors for schizophrenia) and/or study design [34, 35]. Therefore, our findings are extremely important as health planners need local data on schizophrenia rates to assist in planning and implementing effective interventions [34]. According to the Ministry of Health, Labour and Welfare patient survey [36], the estimated number of patients with schizophrenia in Japan in 2017 was 793,000, with 639,000 outpatients and 154,000 inpatients. Our estimation of 0.59% was close to the prevalence rate of outpatient adult schizophrenia patients in Japan. This is understandable as institutionalized patients may not have access to online surveys. In addition, most hospitalized patients were older, which corroborates with the relatively younger average age (42.7 years old) of patients with schizophrenia in this study.

On the other hand, a recently published systematic review and meta-analysis reported a pooled estimate of the prevalence of comorbid major depressive disorder in schizophrenia to be 32.6% [15]. Notably, in our study, 47.8% (85 of 178) and 34.3% (61 of 178) of schizophrenia patients had PHQ-9 score at least 10 and 14, respectively.

The indirect cost of schizophrenia estimated in this study is 1.07 trillion Japanese yen. This cost is lower than the estimated value in a previous study, which revealed an estimation of 1.85 trillion Japanese yen morbidity and mortality cost in Japan in 2008 [10]. One possible explanation for the discrepancy is differences in employment rate and wage calculations between the two studies. In our study, the overall employment rate was 51.1% (Full-time: 20.8%, Part-time: 20.2%, Self-employed: 10.1%) (Supplementary Table 4); in contrast, the employment rate reported in *Sado* et al. was much lower, ranging from 3.8 to 30.6% across gender and age groups [10]. However, it is worth noting that owning to the self-reported nature of the NHWS, it is possible that some respondents might have counted their rehabilitation/other general hardship training as a part-time employment. In addition, although both studies calculated average wage for full-time and part-time employees separately, *Sado* et al. did not separate wage calculation for regular and non-regular (e.g., self-employment) employees.

The inclusion of inpatients could be an alternative explanation for the discrepancy in indirect costs estimated in *Sado* et al. and this study. Inpatients are less likely to be included in our study given that they may not have access to online surveys. According to the Labour and Welfare patient survey conducted by the Ministry of Health in 2017, there were about 154,000 inpatients with schizophrenia in Japan [36]. Using the average annual income (around 5 million yen) reported by the Ministry of Health Labor and Welfare [28] as a proxy, the estimated indirect costs for 154,000 inpatients is about 770 billion yen. This estimated value closely resembles the discrepancy of 780 billion yen (i.e., 1.85 vs. 1.07 trillion) between *Sado* et al and this study. Taken together, this difference in employment rate, the inclusion of inpatients, and wage calculation could potentially lead to the discrepancy in the indirect costs related to productivity loss due to unemployment.

### Patient characteristics

Schizophrenia patients were younger compared to the general population in Japan where around 28% were above 65 years old [37]. This is consistent with global findings where approximately 70.8% of schizophrenia cases occurred in the 25–54 years age group [2]. It is worth noting that, however, the average age of schizophrenia patients in these studies are lower owing to the fact that inpatients, many of whom are elderly over 75 years old [4], may not be included in the study population. Notably, one-third of the patients were overweight. It is important to notice that metabolic syndrome is more likely to occur in schizophrenia patients and psychiatrists should take note of the potential antipsychotic-induced metabolic adverse effects [38, 39]. Additionally, less than half presented at least moderate depressive symptoms (47.8%) and experienced sleep disturbances (49.4%) or anxiety problems (37.1%). The prevalence of comorbid depressive symptoms in our study is aligned to the prevalence reported in the systematic literature review of *Etchecopar-Etchart* et al. (2021) which ranged from 16.3 to 69% [15]. When compared to the data demonstrated by the meta-analysis of *McEnery* et al., our rates on anxiety symptoms are greater, possibly due to demographic characteristics, socioeconomic status, cultural factors, differences in assessment and diagnostic measures, duration of illness and mixed population [17]. Also, it is important to state the limitation for the diagnosis of anxiety once the disturbance is not better explained by another mental disorder, in this case schizophrenia patients [40]. However, our study reported almost identical rates (38.3%) to the data evidenced by the meta-analysis of *Achim* et al. [16]. Finally, the rates of sleep disturbances in our study are similar to those found by *Wang* et al. (58%), which is much higher than the general population [41, 42].

### Health outcomes between schizophrenia patients with milder and more severe depressive symptoms

Our study demonstrated that schizophrenia patients with more severe depressive symptoms were younger and were more often not married in Japan. The unadjusted and adjusted analysis showed that schizophrenia patients with more severe depressive symptoms, compared to those with milder depressive symptoms, had lower HRQoL, higher levels of absenteeism, presenteeism, total work productivity and activity impairment, and twice more indirect costs. We also found an association between severity of depressive symptoms and health outcomes. Depressive symptoms, such as social withdrawal and reduction in work and activities [9], can have severely negative impact on patients with schizophrenia and thus poses a challenge for their social reintegration. Moreover, the psychopathological, social, and health impairment posed by schizophrenia can be worsened by depressive symptoms, which can lead to degradation in patient wellbeing [9, 43]. Last but not least, it has been reported that usage of relapse-related mental health services is higher in schizophrenia patients with severe depressive symptoms and they also had a significantly higher rate of ‘suicidality’ than those without depressive symptoms [6, 9, 44].

### Health outcomes between schizophrenia patients with and without experiencing sleep disturbances

Our study found that schizophrenia patients who experienced sleep disturbances had significantly severe depressive symptoms and consequently, more physician visits and hospitalizations in the past 6 months, compared to patients who did not experience sleep disturbances. Also, schizophrenia patients who experienced sleep disturbances had lower HRQoL, higher levels of absenteeism, presenteeism, total work productivity, and activity impairment, and twice more indirect costs, compared to schizophrenia patients who did not experience sleep disturbances.

*Fang* et al. similarly demonstrated that schizophrenia patients had sleep characteristics significantly different from healthy controls, such as longer sleep onset latency and an average length of awakening, and higher number of awakening and total activity counts [45]. A possible explanation is that these patients have a delta wave deficit reflecting a possible thalamocortical dysfunction [46] or the antipsychotic used [44].

The evidence of our study of lower HRQoL in schizophrenia patients with sleep disturbances is similar to previous studies [47, 48]. Sleep disturbances in schizophrenia patients were reportedly associated with lower HRQoL, depressive symptoms, overall distress, medications’ adverse events and mental and somatic discomfort than those who had no sleep complaints [47], and reduced coping strategies to manage a stressful event [48]. Besides, more than half of schizophrenia patients had sleep disturbances and it was significantly associated with depression and coping mechanisms, and these factors were related to impaired quality of life [41]. Those impaired mechanisms may be explained by the cognitive and memory deficit faced by these patients, which lead to high rigidity, consequently harmful attitudes and impulsive solutions, such as self-injury, influencing sleep quality [49].

It is well known that sleep quality influences work productivity and daily activities [50, 51] and in schizophrenia patients, this had a profound impact on daytime dysfunctions and disability [52]. A study demonstrating the influence of sleep in business found that impaired employee sleep resulted in increased disease burden, health care utilization, absenteeism, and presenteeism, reducing workplace efficiency [53]. Schizophrenia patients have poor sleep quality, and this could impact work productivity levels. Consequently, the indirect costs expressed by morbidity (productivity loss, absenteeism, and presenteeism) increases due to the disease burden, which could also influence the total costs of the disease.

In Australia, the total annual financial cost of inadequate sleep was estimated at US$17.9 billion, of which 68% was comprised of productivity losses largely due to absenteeism and presenteeism [54]. A study in Japan found that subjective sleep quality, daytime dysfunction, use of sleep medicine and sleep disturbances were important variables affecting productivity loss among office employees [51].

### Health outcomes between schizophrenia patients with and without experiencing anxiety problems

Our study evidenced that schizophrenia patients who experienced anxiety problems had more physician visits and hospitalizations in the past 6 months, compared to patients who did not experience anxiety problems. In addition, these patients who experienced anxiety problems had lower HRQoL, higher levels of absenteeism, presenteeism, total work productivity, and activity impairment, and 1.7-fold more indirect costs, compared to schizophrenia patients who did not experience anxiety conditions.

The major clinical problem with schizophrenia and comorbid social anxiety is marked by the fear of acting in a way that may be humiliating or embarrassing [55]. A study with Veterans Health Administration patients with schizophrenia demonstrated that comorbid anxiety disorders were associated with higher rates of psychiatric and medical hospitalization, and increased utilization of outpatient mental health services [56].

Role functioning and quality of life can be seriously affected by social anxiety [57]. Anxiety disorders are associated with increase in level of depression and suicidality, utilization of medical service, and impairment to cognitive and quality of life [14]. Previous studies have reported critical impacts of comorbid social anxiety on social functioning and HRQoL in schizophrenia patients [17]. A worsening in social anxiety and general psychiatric symptoms were associated with a decrease in patient functioning, likely owing to exacerbation of general severity and negative symptoms [14]. The results from the *Aikawa* et al. study revealed that social anxiety in patients with schizophrenia was significantly correlated with the age of onset, duration of untreated psychosis (DUP), social functioning, psychiatric symptoms, and HRQoL. Furthermore, based on a systematic review, social functioning, gender, age of onset, and DUP were identified as important variables associated with the severity of social anxiety symptoms [8].

## Limitations

There are several limitations to our study. Data used in this study were collected from an online survey and was subject to selection bias towards those with Internet access. In addition, the survey is likely to under-represent less healthy elderly people, institutionalized patients, and those with severe comorbidities. It should be noted that a sizable number of patients with schizophrenia were hospitalized [36] and may not have access to online surveys, and thus the prevalence of schizophrenia as well as morbidity cost in this study were likely underestimated. Also, the sample size for schizophrenia patients in this study is not large, which could impose limitations on the interpretation and generalizability of the results. The NHWS is cross-sectional, and thus no causal relationship between diseases and health outcomes could be assumed. In addition, all data used in this study were self-reported and no verification of physician diagnosis and experience of comorbidities was done via other data sources. As many patients with schizophrenia were not aware of their condition, which led to further underestimation of the prevalence.

## Conclusions

Our study results demonstrated that schizophrenia patients with more severe depressive symptoms, sleep disturbances, or anxiety problems experienced significantly poorer quality of life, greater work productivity and activity impairment, and higher indirect costs. The results highlight the significance of screening and treating the relevant comorbid conditions among patients with schizophrenia to improve their overall quality of life.

## Supplementary Information

**Additional file 1.**

**Additional file 2.**

## Data Availability

Study data to support our findings are available from Cerner Enviza, but availability of the data is restricted and was used under license for this study and are not publicly available. Data are however available from the authors upon reasonable request and with permission of Cerner Enviza.

## Abbreviations

BMI: Body Mass Index
CCI: Charlson Comorbidity Index
CI: Confidence Interval
EQ-5D-5L: EuroQoL 5-dimensions Scale
ER: Emergency Room
GLM: Generalized Linear Model
HCP: Healthcare Provider
HCRU: Healthcare Resources Utilization
HRQoL: Health-related Quality of Life
MCS: Mental Component Summary
NHWS: National Health and Wellness Survey
PCS: Physical Component Summary
PHQ-9: Patient Health Questionnaire-9
QoL: Quality of Life
RCS: Role Component Summary
SD: Standard Deviation
SF-12v2: Short Form 12-Item (version 2) Health Survey
VAS: Visual Analog Scale
WPAI: Work Productivity and Activity Impairment
YLD: years of life lived with disability
