# Supplementary material for: Burden of schizophrenia among Japanese patients: a cross-sectional National Health and Wellness Survey
Source: BMC Psychiatry. 2022 Jun 18;22:410. doi: 10.1186/s12888-022-04044-5 (PMC9206739; doi:10.1186/s12888-022-04044-5)
Supplement: Supplementary file 1 — Additional file 1. [file 12888_2022_4044_MOESM1_ESM.docx]

# **Supplementary Material**

**Supplementary Table 1. Demographic and general health characteristics of schizophrenia patients with PHQ-9<14 vs. PHQ-9≥14 in Japan**

|  | | **Schizophrenia patients and**  **PHQ-9<14  N=117** | | | **Schizophrenia patients and**  **PHQ-9≥14  N=61** | |  |
| --- | --- | --- | --- | --- | --- | --- | --- |
| **Continuous Variables** |  | **Mean** | **SD** | **Mean** | | **SD** | **P-value** |
| Age | | 45.09 | 14.34 | 38.11 | | 13.41 | 0.002 |
| Charlson Comorbidity Index | | 0.52 | 3.11 | 0.61 | | 2.33 | 0.851 |
| Healthcare resource utilisation | *No. of physician visits in the past 6 months* | 11.03 | 10.77 | 14.23 | | 14.20 | 0.094 |
|  | *No. of ER visits in the past 6 months* | 0.11 | 0.85 | 0.15 | | 0.51 | 0.759 |
|  | *No. of hospitalizations in the past 6 months* | 0.26 | 1.25 | 0.20 | | 0.57 | 0.686 |
| **Categorical Variables** |  | **N** | **%** | **N** | | **%** | **P-value** |
| Age group | *18-64* | 102 | 87.2% | 57 | | 93.4% | 0.199 |
|  | *≥65* | 15 | 12.8% | 4 | | 6.6% |  |
| Gender | *Male* | 61 | 52.1% | 28 | | 45.9% | 0.430 |
|  | *Female* | 56 | 47.9% | 33 | | 54.1% |  |
| Marital Status | *Married or living with partner* | 40 | 34.2% | 12 | | 19.7% | 0.043 |
|  | *Not married/Decline to answer* | 77 | 65.8% | 49 | | 80.3% |  |
| Level of Education | *University degree* | 39 | 33.3% | 19 | | 31.1% | 0.768 |
|  | *Not/Decline to answer* | 78 | 66.7% | 42 | | 68.9% |  |
| Employment Status | *Currently employed* | 62 | 53.0% | 29 | | 47.5% | 0.490 |
|  | *Not* | 55 | 47.0% | 32 | | 52.5% |  |
| Household Income | *<¥3,000,000* | 48 | 41.0% | 22 | | 36.1% | 0.300 |
|  | *¥3,000,000 to <¥5,000,000* | 29 | 24.8% | 15 | | 24.6% |  |
|  | *¥5,000,000 to <¥8,000,000* | 17 | 14.5% | 7 | | 11.5% |  |
|  | *¥8,000,000 or more* | 13 | 11.1% | 5 | | 8.2% |  |
|  | *Decline to answer* | 10 | 8.5% | 12 | | 19.7% |  |
| Body Mass Index | *Underweight (BMI < 18.5)* | 13 | 11.1% | 8 | | 13.1% | 0.176 |
|  | *Normal (BMI >=18.5 & <25)* | 56 | 47.9% | 37 | | 60.7% |  |
|  | *Overweight (BMI >=25)* | 44 | 37.6% | 13 | | 21.3% |  |
|  | *Decline to answer* | 4 | 3.4% | 3 | | 4.9% |  |
| Smoking Status | *Never* | 63 | 53.8% | 29 | | 47.5% | 0.152 |
|  | *Former* | 29 | 24.8% | 11 | | 18.0% |  |
|  | *Current* | 25 | 21.4% | 21 | | 34.4% |  |
| Alcohol Use | *≤once per week* | 94 | 80.3% | 49 | | 80.3% | 0.998 |
|  | *≥2-3 times per week* | 23 | 19.7% | 12 | | 19.7% |  |
| Vigorous Exercise in Past 30 Days | *0-11 times* | 97 | 82.9% | 54 | | 88.5% | 0.321 |
|  | *≥12 times* | 20 | 17.1% | 7 | | 11.5% |  |
| Insurance type | *National health insurance* | 56 | 47.9% | 33 | | 54.1% | 0.545 |
|  | *Social insurance* | 39 | 33.3% | 17 | | 27.9% |  |
|  | *Late stage elderly insurance* | 6 | 5.1% | 1 | | 1.6% |  |
|  | *Other/No insurance* | 16 | 13.7% | 10 | | 16.4% |  |
| Out of pocket payment for own prescription medications in an average month | *¥0* | 23 | 19.7% | 12 | | 19.7% | 0.378 |
|  | *¥1 to ¥999* | 20 | 17.1% | 5 | | 8.2% |  |
|  | *¥1,000 to ¥1,999* | 16 | 13.7% | 5 | | 8.2% |  |
|  | *¥2,000 to ¥2,999* | 9 | 7.7% | 7 | | 11.5% |  |
|  | *¥3,000 to ¥4,999* | 14 | 12.0% | 10 | | 16.4% |  |
|  | *¥5,000 to ¥9,999* | 13 | 11.1% | 4 | | 6.6% |  |
|  | *¥10,000 to ¥49,999* | 5 | 4.3% | 6 | | 9.8% |  |
|  | *¥50,000 to ¥99,999* | 0 | 0.0% | 1 | | 1.6% |  |
|  | *¥100,000 or more* | 2 | 1.7% | 1 | | 1.6% |  |
|  | *Don't know* | 15 | 12.8% | 10 | | 16.4% |  |
| Region | *Hokkaido* | 5 | 4.3% | 3 | | 4.9% | 0.973 |
|  | *Tohoku* | 10 | 8.5% | 6 | | 9.8% |  |
|  | *Kanto* | 36 | 30.8% | 21 | | 34.4% |  |
|  | *Chubu* | 17 | 14.5% | 9 | | 14.8% |  |
|  | *Kansai/Kinki* | 25 | 21.4% | 9 | | 14.8% |  |
|  | *Chugoku* | 9 | 7.7% | 4 | | 6.6% |  |
|  | *Shikoku* | 5 | 4.3% | 2 | | 3.3% |  |
|  | *Kyushu/Okinawa* | 10 | 8.5% | 7 | | 11.5% |  |

**Supplementary Table 2. Unadjusted means of health outcomes for schizophrenia patients with PHQ-9<14 vs. PHQ-9≥14 in Japan**

|  | | **Schizophrenia patients and**  **PHQ-9<14  N=117** | | | **Schizophrenia patients and**  **PHQ-9≥14  N=61** | | |  |
| --- | --- | --- | --- | --- | --- | --- | --- | --- |
| **Health outcomes (Unadjusted)** | | **N** | **Mean** | **SD** | **N** | **Mean** | **SD** | **P-value** |
| HRQoL | *MCS (Japanese norm)* | 117 | 48.88 | 11.21 | 61 | 39.00 | 9.19 | < 0.001 |
|  | *PCS (Japanese norm)* | 117 | 46.42 | 13.11 | 61 | 45.81 | 15.75 | 0.783 |
|  | *RCS (Japanese norm)* | 117 | 39.55 | 15.88 | 61 | 23.07 | 15.39 | < 0.001 |
|  | *EQ-5D index* | 117 | 0.77 | 0.18 | 61 | 0.59 | 0.18 | < 0.001 |
|  | *EQ-5D VAS* | 117 | 64.17 | 22.79 | 61 | 41.26 | 25.88 | < 0.001 |
| WPAI | *Absenteeism %* | 59 | 13.37 | 23.10 | 28 | 25.25 | 33.05 | 0.056 |
|  | *Presenteeism %* | 61 | 30.98 | 28.85 | 27 | 65.93 | 26.78 | < 0.001 |
|  | *Total work impairment %* | 58 | 37.84 | 30.68 | 27 | 70.22 | 27.22 | < 0.001 |
|  | *Total activity impairment %* | 117 | 33.76 | 27.94 | 61 | 63.28 | 26.94 | < 0.001 |
| Indirect cost (in thousand yen) | | 58 | 1,423.12 | 1,191.21 | 27 | 2,542.43 | 1,257.37 | < 0.001 |

**Supplementary Table 3. Demographic and general health characteristics of schizophrenia patients with and without experience of sleep disturbances and anxiety problems in Japan**

|  | | **Schizophrenia patients with experience of sleep disturbances  N=88 (1)** | | | **Schizophrenia patients without experience of sleep disturbances  N=90 (2)** | | **Schizophrenia patients with experience of anxiety problems  N=66 (3)** | | **Schizophrenia patients without experience of anxiety problems  N=112 (4)** | | **P-value** | |
| --- | --- | --- | --- | --- | --- | --- | --- | --- | --- | --- | --- | --- |
| **Continuous Variables** |  | **Mean** | **SD** | **Mean** | | **SD** | **Mean** | **SD** | **Mean** | **SD** | **(1) vs. (2)** | **(3) vs. (4)** |
| Age | | 41.70 | 14.66 | 43.67 | | 14.10 | 40.48 | 15.81 | 44.00 | 13.36 | 0.364 | 0.115 |
| Charlson Comorbidity Index | | 0.95 | 4.01 | 0.16 | | 0.45 | 1.23 | 4.60 | 0.15 | 0.49 | 0.062 | 0.015 |
| Healthcare resource utilisation | *No. of physician visits in the past 6 months* | 16.64 | 14.36 | 7.71 | | 7.11 | 16.09 | 14.50 | 9.79 | 9.80 | < 0.001 | < 0.001 |
|  | *No. of ER visits in the past 6 months* | 0.13 | 0.45 | 0.12 | | 0.96 | 0.17 | 0.51 | 0.10 | 0.86 | 0.980 | 0.557 |
|  | *No. of hospitalizations in the past 6 months* | 0.41 | 1.47 | 0.08 | | 0.31 | 0.47 | 1.62 | 0.11 | 0.47 | 0.038 | 0.028 |
| **Categorical Variables** |  | **N** | **%** | **N** | | **%** | **N** | **%** | **N** | **%** | **(1) vs. (2)** | **(3) vs. (4)** |
| Age group | *18-64* | 79 | 89.8% | 80 | | 88.9% | 58 | 87.9% | 101 | 90.2% | 0.849 | 0.631 |
|  | *≥65* | 9 | 10.2% | 10 | | 11.1% | 8 | 12.1% | 11 | 9.8% |  |  |
| Gender | *Male* | 44 | 50.0% | 45 | | 50.0% | 32 | 48.5% | 57 | 50.9% | 1.000 | 0.756 |
|  | *Female* | 44 | 50.0% | 45 | | 50.0% | 34 | 51.5% | 55 | 49.1% |  |  |
| Marital Status | *Married or living with partner* | 24 | 27.3% | 28 | | 31.1% | 22 | 33.3% | 30 | 26.8% | 0.573 | 0.353 |
|  | *Not married/Decline to answer* | 64 | 72.7% | 62 | | 68.9% | 44 | 66.7% | 82 | 73.2% |  |  |
| Level of Education | *University degree* | 34 | 38.6% | 24 | | 26.7% | 22 | 33.3% | 36 | 32.1% | 0.088 | 0.870 |
|  | *Not/Decline to answer* | 54 | 61.4% | 66 | | 73.3% | 44 | 66.7% | 76 | 67.9% |  |  |
| Employment Status | *Currently employed* | 48 | 54.5% | 43 | | 47.8% | 37 | 56.1% | 54 | 48.2% | 0.366 | 0.312 |
|  | *Not* | 40 | 45.5% | 47 | | 52.2% | 29 | 43.9% | 58 | 51.8% |  |  |
| Household Income | *<¥3,000,000* | 37 | 42.0% | 33 | | 36.7% | 27 | 40.9% | 43 | 38.4% | 0.051 | 0.409 |
|  | *¥3,000,000 to <¥5,000,000* | 19 | 21.6% | 25 | | 27.8% | 12 | 18.2% | 32 | 28.6% |  |  |
|  | *¥5,000,000 to <¥8,000,000* | 8 | 9.1% | 16 | | 17.8% | 8 | 12.1% | 16 | 14.3% |  |  |
|  | *¥8,000,000 or more* | 14 | 15.9% | 4 | | 4.4% | 9 | 13.6% | 9 | 8.0% |  |  |
|  | *Decline to answer* | 10 | 11.4% | 12 | | 13.3% | 10 | 15.2% | 12 | 10.7% |  |  |
| Body Mass Index | *Underweight (BMI < 18.5)* | 12 | 13.6% | 9 | | 10.0% | 11 | 16.7% | 10 | 8.9% | 0.745 | 0.261 |
|  | *Normal (BMI >=18.5 & <25)* | 43 | 48.9% | 50 | | 55.6% | 32 | 48.5% | 61 | 54.5% |  |  |
|  | *Overweight (BMI >=25)* | 30 | 34.1% | 27 | | 30.0% | 22 | 33.3% | 35 | 31.3% |  |  |
|  | *Decline to answer* | 3 | 3.4% | 4 | | 4.4% | 1 | 1.5% | 6 | 5.4% |  |  |
| Smoking Status | *Never* | 41 | 46.6% | 51 | | 56.7% | 29 | 43.9% | 63 | 56.3% | 0.188 | 0.048 |
|  | *Former* | 19 | 21.6% | 21 | | 23.3% | 13 | 19.7% | 27 | 24.1% |  |  |
|  | *Current* | 28 | 31.8% | 18 | | 20.0% | 24 | 36.4% | 22 | 19.6% |  |  |
| Alcohol Use | *≤once per week* | 70 | 79.5% | 73 | | 81.1% | 52 | 78.8% | 91 | 81.3% | 0.793 | 0.690 |
|  | *≥2-3 times per week* | 18 | 20.5% | 17 | | 18.9% | 14 | 21.2% | 21 | 18.8% |  |  |
| Vigorous Exercise in Past 30 Days | *0-11 times* | 74 | 84.1% | 77 | | 85.6% | 59 | 89.4% | 92 | 82.1% | 0.785 | 0.193 |
|  | *≥12 times* | 14 | 15.9% | 13 | | 14.4% | 7 | 10.6% | 20 | 17.9% |  |  |
| Insurance type | *National health insurance* | 40 | 45.5% | 49 | | 54.4% | 32 | 48.5% | 57 | 50.9% | 0.195 | 0.173 |
|  | *Social insurance* | 29 | 33.0% | 27 | | 30.0% | 19 | 28.8% | 37 | 33.0% |  |  |
|  | *Late stage elderly insurance* | 2 | 2.3% | 5 | | 5.6% | 1 | 1.5% | 6 | 5.4% |  |  |
|  | *Other/No insurance* | 17 | 19.3% | 9 | | 10.0% | 14 | 21.2% | 12 | 10.7% |  |  |
| Out of pocket payment for own prescription medications in an average month | *¥0* | 19 | 21.6% | 16 | | 17.8% | 11 | 16.7% | 24 | 21.4% | 0.017 | 0.094 |
|  | *¥1 to ¥999* | 5 | 5.7% | 20 | | 22.2% | 3 | 4.5% | 22 | 19.6% |  |  |
|  | *¥1000 to ¥1999* | 7 | 8.0% | 14 | | 15.6% | 8 | 12.1% | 13 | 11.6% |  |  |
|  | *¥2000 to ¥2999* | 8 | 9.1% | 8 | | 8.9% | 6 | 9.1% | 10 | 8.9% |  |  |
|  | *¥3000 to ¥4999* | 16 | 18.2% | 8 | | 8.9% | 10 | 15.2% | 14 | 12.5% |  |  |
|  | *¥5000 to ¥9,999* | 9 | 10.2% | 8 | | 8.9% | 6 | 9.1% | 11 | 9.8% |  |  |
|  | *¥10,000 to ¥49,999* | 9 | 10.2% | 2 | | 2.2% | 7 | 10.6% | 4 | 3.6% |  |  |
|  | *¥50,000 to ¥99,999* | 1 | 1.1% | 0 | | 0.0% | 1 | 1.5% | 0 | 0.0% |  |  |
|  | *¥100,000 or more* | 2 | 2.3% | 1 | | 1.1% | 2 | 3.0% | 1 | 0.9% |  |  |
|  | *Don't know* | 12 | 13.6% | 13 | | 14.4% | 12 | 18.2% | 13 | 11.6% |  |  |
| Region | *Hokkaido* | 5 | 5.7% | 3 | | 3.3% | 5 | 7.6% | 3 | 2.7% | 0.033 | 0.557 |
|  | *Tohoku* | 7 | 8.0% | 9 | | 10.0% | 7 | 10.6% | 9 | 8.0% |  |  |
|  | *Kanto* | 28 | 31.8% | 29 | | 32.2% | 22 | 33.3% | 35 | 31.3% |  |  |
|  | *Chubu* | 8 | 9.1% | 18 | | 20.0% | 8 | 12.1% | 18 | 16.1% |  |  |
|  | *Kansai/Kinki* | 15 | 17.0% | 19 | | 21.1% | 11 | 16.7% | 23 | 20.5% |  |  |
|  | *Chugoku* | 7 | 8.0% | 6 | | 6.7% | 4 | 6.1% | 9 | 8.0% |  |  |
|  | *Shikoku* | 3 | 3.4% | 4 | | 4.4% | 1 | 1.5% | 6 | 5.4% |  |  |
|  | *Kyushu/Okinawa* | 15 | 17.0% | 2 | | 2.2% | 8 | 12.1% | 9 | 8.0% |  |  |

**Supplementary Table 4. Employment status of schizophrenia patients in Japan**

| **Employment status** | **Schizophrenia patients** | |
| --- | --- | --- |
|  | **N** | **%** |
| Employed full time | 37 | 20.8% |
| Self-employed | 18 | 10.1% |
| Employed part time | 36 | 20.2% |
| Homemaker | 28 | 15.7% |
| Retired | 4 | 2.2% |
| Student | 1 | 0.6% |
| Long-Term Disability: Long term leave of absence due to illness of your own (more than 3 months) | 9 | 5.1% |
| Not employed, but looking for work | 6 | 3.4% |
| Not employed and not looking for work | 37 | 20.8% |
| Short-Term Disability: Short team leave of absence due to illness of your own (less than 3 months) | 2 | 1.1% |
| **Total** | **178** | **100.0**% |

**Supplementary Figure 1. Associations between the PHQ-9 score and selected health outcomes indices.**


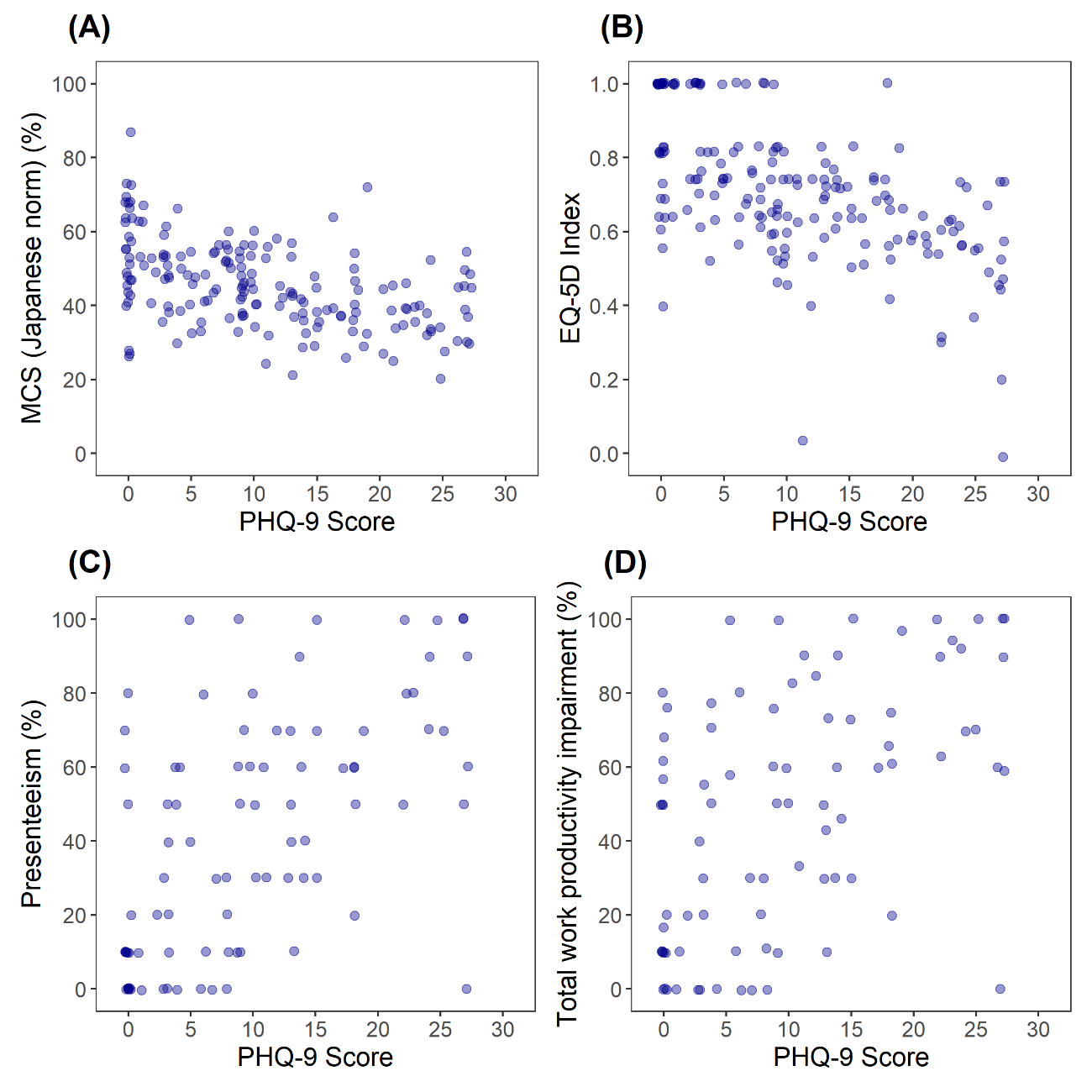


Each panel illustrates the association between PHQ-9 score and one of the following indices: (A) MCS (Japanese norm); (B) EQ-5D index; (C) presenteeism; (D) total work productivity impairment. Data points are jittered to minimize overlapping.
