# Supplementary material for: Burden of schizophrenia among Japanese patients: a cross-sectional National Health and Wellness Survey
Source: BMC Psychiatry. 2022 Jun 18;22:410. doi: 10.1186/s12888-022-04044-5 (PMC9206739; doi:10.1186/s12888-022-04044-5)
Supplement: Supplementary file 2 — Additional file 2. [file 12888_2022_4044_MOESM2_ESM.docx]

**Estimation of the morbidity cost of schizophrenia**

# **Methods**

Morbidity costs refer to the wage losses by people who are unable to commit to work because of illness (1–3). In this additional analysis, morbidity costs included both losses caused by absenteeism and presenteeism for the employed (work productivity loss cost) and losses caused by unemployment for the unemployed (unemployment cost). A human capital approach was applied to estimate the annual morbidity cost of adult patients with schizophrenia using available data from National Health and Wellness Survey (NHWS) Japan 2019 and Labour Force Survey Japan 2019 (4). Gender and age-specific population statistics, labour status and earnings statistics of Japan were retrieved from the Labour Force Survey Japan 2019 (4).

## ***Work productivity cost calculation***

For this additional cost estimation, work productivity loss costs were estimated separately for full-time and part-time workers due to the wage differences between full-time and part-time employment. The total work productivity loss cost was the sum of all gender-age-specific work productivity loss costs. The gender and age-specific work productivity loss cost for full-time workers was calculated by a product of estimated number of female or male schizophrenia patients at an age group, employment rate of the patients, their full-time employment ratio, overall work productivity impairment and corresponding average yearly earnings for full-time employees in Japan in 2019 (Supplementary Table 5). The number of female or male patients at each age group was estimated by multiplying the gender and age-specific prevalence of schizophrenia by using NHWS 2019 data with the population statistics.

The employment rate of the patients and the average yearly earnings were gender and age-specific, and the full-time employment ratio and overall work productivity impairment were gender-specific. Similarly, the calculation can be applied to estimate the gender and age-specific work productivity loss costs for part-time workers (Supplementary Table 5).

## ***Unemployment cost calculations***

The unemployment costs were assumed to occur when there was a gap between the employment rate of general population and the employment rate of schizophrenia patients (2).

Unemployment costs were also estimated separately for full-time and part-time workers. The total unemployment cost was the sum of all gender and age-specific unemployment costs. The gender and age-specific unemployment cost for full-time workers was calculated by multiplying estimated number of female or male schizophrenia patients at an age group, with the employment rate difference between the general population and schizophrenia patients, with full-time employment ratio and corresponding average yearly earnings for full-time employees in Japan in 2019 (Supplementary Table 5). Similarly, the calculation can be applied to estimate the gender and age-specific unemployment costs for part-time workers (Supplementary Table 5).

# **Results**

Work productivity loss costs and unemployment costs for both full-time and part-time workers among patients with schizophrenia for each age and gender group are displayed in detail in Supplementary Table 6.

The total work productivity loss cost due to schizophrenia was estimated to be Japanese yen (JP¥) 522 billion, while the total unemployment cost was estimated to be JP¥ 552 billion. This brings the total national morbidity costs of schizophrenia in Japan to be approximately JP¥ 1,074 billion (Supplementary Table 7).

**Supplementary Table 5. Morbidity cost calculation formula for male/female at each age group in Japan in 2019**

| **Morbidity cost** | **Work productivity loss cost** | For full-time workers | Estimated No. of male/female patients at an age group ×  gender and age-specific employment rate of the patients ×  gender-specific full-time employment ratio of the patients ×  gender-specific full-time work productivity impairment of the patients ×  gender and age-specific average yearly earnings for full-time employees in Japan in 2019 |
| --- | --- | --- | --- |
|  |  | For part-time workers | Estimated No. of male/female patients at an age group × gender and age-specific employment rate of the patients ×  gender-specific part-time employment ratio of the patients ×  gender-specific part-time work productivity impairment of the patients ×  gender and age-specific average yearly earnings for part-time employees in Japan in 2019 |
|  | **Unemployment cost** | For full-time workers | Estimated No. of male/female patients at an age group × (gender and age-specific employment rate in Japan in 2019 - gender and age-specific employment rate of the patients) ×  gender-specific full-time employment ratio of the patients ×  gender and age-specific average yearly earnings for full-time employees in Japan in 2019 |
|  |  | For part-time workers | Estimated No. of male/female patients at an age group × (gender and age-specific employment rate in Japan in 2019 - gender and age-specific employment rate of the patients) ×  gender-specific part-time employment ratio of the patients ×  gender and age-specific average yearly earnings for part-time employees in Japan in 2019 |

**Supplementary Table 6. Morbidity cost calculation for schizophrenia patients**

| **Schizophrenia** | | | | | | | | | | | | | | | | |
| --- | --- | --- | --- | --- | --- | --- | --- | --- | --- | --- | --- | --- | --- | --- | --- | --- |
| **Male** | | | | | | | | | | | | | | | | |
| **Age group** | **Prevalence rate A** | **Employment rate B** | **No. of patients C = A x Population** | **No. of employed patients D = B x C** | **Full-time employment rate (%)  E** | **Part-time employment rate (%)  F** | **Full-time employed overall work impairment (%)**  **G** | **Part-time employed overall work impairment (%)**  **H** | **Yearly earnings of full-time employed (Yen)  I** | **Yearly earnings of part-time employed (Yen)**  **J** | **Full-time employed overall work impairment cost (Yen)**  **K＝D×E×G×I** | **Part-time employed overall work impairment cost (Yen)**  **L＝D×F×H×J** | **Employment rate in the general population (%)  M** | **Excessive unemployment rate of patients (%)  N＝M－B** | **Full-time labour unemployment cost (Yen) O＝C×E×I×N** | **Part-time labour unemployment cost (Yen) P＝C×F×J×N** |
| **20-24** | 0.47% | 50.0% | 14,038 | 7,019 | 45.0% | 55.0% | 47.3% | 50.3% | 3,068,200 | 2,304,200 | 4,583,728,747 | 4,474,169,532 | 73.2% | 23.2% | 4,505,068,310 | 4,135,119,484 |
| **25-29** | 1.14% | 50.0% | 34,393 | 17,196 | 45.0% | 55.0% | 47.3% | 50.3% | 3,884,500 | 2,635,000 | 14,218,229,000 | 12,535,683,499 | 90.4% | 40.4% | 24,305,682,463 | 20,151,319,523 |
| **30-34** | 0.50% | 50.0% | 17,905 | 8,953 | 45.0% | 55.0% | 47.3% | 50.3% | 4,570,900 | 2,793,300 | 8,710,146,318 | 6,918,283,136 | 92.8% | 42.8% | 15,747,377,431 | 11,761,813,092 |
| **35-39** | 1.43% | 64.7% | 52,660 | 34,074 | 45.0% | 55.0% | 47.3% | 50.3% | 5,181,800 | 2,861,500 | 37,581,868,133 | 26,974,194,704 | 93.5% | 28.8% | 35,330,454,545 | 23,845,833,333 |
| **40-44** | 1.13% | 71.4% | 51,781 | 36,986 | 45.0% | 55.0% | 47.3% | 50.3% | 5,719,200 | 2,930,700 | 45,024,597,863 | 29,987,685,185 | 95.0% | 23.6% | 31,397,993,231 | 19,664,737,228 |
| **45-49** | 0.91% | 81.3% | 43,628 | 35,448 | 45.0% | 55.0% | 47.3% | 50.3% | 6,258,400 | 3,080,800 | 47,220,090,994 | 30,212,316,666 | 94.0% | 12.7% | 15,630,034,694 | 9,403,949,887 |
| **50-54** | 0.80% | 90.0% | 35,040 | 31,536 | 45.0% | 55.0% | 47.3% | 50.3% | 6,917,100 | 3,085,500 | 46,430,602,123 | 26,919,242,341 | 93.4% | 3.4% | 3,685,430,880 | 2,009,277,600 |
| **55-59** | 0.37% | 60.0% | 13,903 | 8,342 | 45.0% | 55.0% | 47.3% | 50.3% | 6,842,600 | 3,118,700 | 12,149,528,284 | 7,197,288,038 | 91.8% | 31.8% | 13,622,446,882 | 7,588,531,585 |
| **60-64** | 0.19% | 50.0% | 6,970 | 3,485 | 45.0% | 55.0% | 47.3% | 50.3% | 5,060,100 | 3,731,400 | 3,753,632,499 | 3,597,671,271 | 82.1% | 32.1% | 5,100,072,909 | 4,596,627,045 |
| **65-69** | 0.20% | 60.0% | 8,419 | 5,052 | 45.0% | 55.0% | 47.3% | 50.3% | 4,143,500 | 3,030,600 | 4,455,144,882 | 4,235,260,498 | 59.5% | -0.5% | -73,873,811 | -66,039,223 |
| **≥70** | 0.13% | 50.0% | 14,751 | 7,375 | 45.0% | 55.0% | 47.3% | 50.3% | 3,645,300 | 2,613,900 | 5,722,499,385 | 5,333,330,140 | 24.6% | -25.4% | -6,150,775,187 | -5,390,582,817 |
| **Female** | | | | | | | | | | | | | | | | |
| **20-24** | 1.39% | 66.7% | 39,861 | 26,574 | 32.3% | 67.7% | 47.2% | 46.1% | 2,946,600 | 2,190,800 | 11,937,642,647 | 18,169,592,824 | 75.2% | 8.5% | 3,227,776,139 | 5,030,036,088 |
| **25-29** | 0.95% | 35.3% | 27,013 | 9,534 | 32.3% | 67.7% | 47.2% | 46.1% | 3,579,700 | 2,393,800 | 5,203,132,903 | 7,122,807,760 | 82.3% | 47.0% | 14,691,623,444 | 20,591,935,339 |
| **30-34** | 0.97% | 36.4% | 34,011 | 12,367 | 32.3% | 67.7% | 47.2% | 46.1% | 3,890,300 | 2,418,400 | 7,335,153,777 | 9,334,677,904 | 76.3% | 39.9% | 17,061,336,796 | 22,230,244,714 |
| **35-39** | 0.40% | 0.0% | 14,390 | 0 | 32.3% | 67.7% | 47.2% | 46.1% | 4,087,600 | 2,475,100 | 0 | 0 | 75.5% | 75.5% | 14,341,358,779 | 18,201,232,896 |
| **40-44** | 1.00% | 10.0% | 44,389 | 4,439 | 32.3% | 67.7% | 47.2% | 46.1% | 4,366,400 | 2,485,100 | 2,954,889,360 | 3,442,759,207 | 78.3% | 68.3% | 42,776,763,271 | 51,028,689,809 |
| **45-49** | 0.55% | 66.7% | 25,855 | 17,236 | 32.3% | 67.7% | 47.2% | 46.1% | 4,525,400 | 2,462,500 | 11,891,787,537 | 13,246,815,303 | 80.2% | 13.5% | 5,102,676,480 | 5,819,738,182 |
| **50-54** | 0.75% | 55.6% | 32,271 | 17,928 | 32.3% | 67.7% | 47.2% | 46.1% | 4,676,200 | 2,441,200 | 12,781,458,238 | 13,659,530,185 | 78.4% | 22.9% | 11,145,889,985 | 12,195,824,948 |
| **55-59** | 0.71% | 42.9% | 26,586 | 11,394 | 32.3% | 67.7% | 47.2% | 46.1% | 4,549,100 | 2,398,700 | 7,902,125,263 | 8,529,811,509 | 73.9% | 31.1% | 12,140,766,741 | 13,417,843,214 |
| **60-64** | 0.30% | 0.0% | 11,842 | 0 | 32.3% | 67.7% | 47.2% | 46.1% | 3,963,600 | 2,539,300 | 0 | 0 | 58.6% | 58.6% | 8,885,703,452 | 11,931,695,306 |
| **65-69** | 0.31% | 0.0% | 14,148 | 0 | 32.3% | 67.7% | 47.2% | 46.1% | 3,640,300 | 2,316,400 | 0 | 0 | 38.6% | 38.6% | 6,414,958,781 | 8,555,716,729 |
| **≥70** | 0.14% | 0.0% | 21,177 | 0 | 32.3% | 67.7% | 47.2% | 46.1% | 3,655,300 | 2,150,300 | 0 | 0 | 11.8% | 11.8% | 2,939,672,390 | 3,624,608,530 |

**Supplementary Table 7. National morbidity cost for schizophrenia in Japan in 2019**

|  |  | **Schizophrenia**  **(in Billion JP**¥**)** |
| --- | --- | --- |
| Work productivity loss cost | *Male* | 388 |
|  | *Female* | 134 |
|  | *Total* | 522 |
| Unemployment cost | *Male* | 241 |
|  | *Female* | 311 |
|  | *Total* | 552 |
| Morbidity Cost | *Male* | 629 |
|  | *Female* | 445 |
|  | *Total* | 1,074 |

# **References**

1. P K, J S. The costs of depression. Int Clin Psychopharmacol. 1993 Jan 1;7(3–4):191–5.

2. Sado M, Inagaki A, Koreki A, Knapp M, Kissane LA, Mimura M, et al. The cost of schizophrenia in Japan. Neuropsychiatr Dis Treat. 2013;9:787–98.

3. Okumura Y, Higuchi T. Cost of depression among adults in Japan. Prim Care Companion CNS Disord. 2011;13(3).

4. 統計局ホームページ/令和元年　労働力調査年報 [Internet]. [cited 2021 Apr 29]. Available from: https://www.stat.go.jp/data/roudou/report/2019/index.html
